# Deep inspiration breath hold real-time tumor-tracking radiation therapy (DBRT) as a novel stereotactic body radiation therapy approach for lung tumors

**DOI:** 10.1038/s41598-024-53020-4

**Published:** 2024-01-29

**Authors:** Hidekazu Tanaka, Taiki Ono, Kazushi Ueda, Masako Karita, Yuki Manabe, Miki Kajima, Tatsuhiro Sera, Koya Fujimoto, Yuki Yuasa, Takehiro Shiinoki

**Affiliations:** Department of Radiation Oncology, Yamaguchi University Graduate School of Medicine, 1-1-1 Minamikogushi, Ube, 755-8505 Japan

**Keywords:** Oncology, Cancer

## Abstract

Radiotherapy with deep inspiration breath hold (DIBH) reduces doses to the lungs and organs at risk. The stability of breath holding and reproducibility of tumor location are higher during expiration than during inspiration; therefore, we developed an irradiation method combining DIBH and real-time tumor-tracking radiotherapy (RTRT) (DBRT). Nine patients were enrolled in this study. Fiducial markers were placed near tumors using bronchoscopy. Treatment planning computed tomography (CT) was performed thrice during DIBH, assisted by spirometer-based device. Each CT scan was fused using fiducial markers. Gross tumor volume (GTV) was contoured for each dataset and summed to create GTVsum; adding a 5-mm margin around GTVsum generated the planning target volume. The prescribed dose was mainly 42 Gy in four fractions. The treatment plan was created using DIBH CT (DBRT-plan), with a similar treatment plan created for expiratory CT for cases for which DBRT could not be performed (conv-plan). Vx defined as the volume of the lung received x Gy, and the mean lung dose, V20, V10, and V5 were evaluated. DBRT was completed in all patients. Mean dose, V20, and V10 were significantly lower in the DBRT-plan than in the conv-plan (all p = 0.003). Mean rates of decrease for mean dose, V20, and V10 were 14.0%, 27.6%, and 19.1%, respectively. No significant difference was observed in V5. We developed DBRT, a stereotactic body radiation therapy performed with the DIBH technique; it combines a spirometer-based breath-hold support system with an RTRT system. All patients who underwent DBRT completed the procedure without any technical or mechanical complications. This is a promising methodology that may significantly reduce lung doses.

## Introduction

Lung tumors can move significantly during respiration. Unless countermeasures are taken, irradiating a tumor that moves with respiration requires that the irradiated area include the entire path of the moving tumor, resulting in a large irradiation volume. Respiratory motion management (RMM) is used to avoid this^1^. RMM plays an important role in stereotactic body radiation therapy (SBRT), which requires high accuracy. Breath hold (BH), one of several RMM techniques, can be performed using the position of the chest or abdominal wall, the shape of the body surface, or the volume of lung ventilation as indicators^2–8^. Methods using ventilation volume as the indicator are more accurate than those using chest or abdominal wall position or body surface shape^9,10^. BH is more stable and shows greater reproducibility of tumor location in expiration than in inspiration^11,12^. Therefore, expiration is often employed when irradiation is performed using the BH technique^13^.

Real-time tumor-tracking radiation therapy (RTRT) is a highly accurate type of RMM^14,15^. We used an RTRT system with a tracking error of < 0.4 mm^16^. This method tracks, in real time, fiducial markers (FMs) placed near the tumor to determine the tumor location: irradiation is performed only when the FM is present at a defined position—called a gating box—in three dimensions. The FM is used as an index; because the marker is placed in the body, it has a higher correlation with the position of the tumor than indexing methods that use external information such as the position of the chest or abdominal wall and the body surface^17^. The speed at which tumors move due to respiration is fast during inspiration and slow during expiration^18^. In RTRT, there is a slight beam-on delay from the time the marker enters the gating box until the start of irradiation^16^. As a result, the distance the tumor moves during the beam-on delay is larger during inspiration than during expiration, resulting in a large margin that needs to be added. Therefore, in RTRT, irradiation is performed at the end of expiration, and irradiation with RMM often involves the expiratory phase. However, during expiration, the lung collapses. This collapse may result in a relative increase in lung dose that leads to more adverse events occurring. The lung dose can be reduced if irradiation is performed during inspiration, especially deep inspiration BH (DIBH). Performing SBRT with DIBH requires high positional accuracy; therefore, a high reproducibility of the tumor location must be ensured. Thus, we developed DIBH RTRT (DBRT), a method of irradiation that uses RTRT during DIBH with ventilation volume as the indicator.

## Methods

### Patients

All procedures performed in this study, which involved human participants, were conducted according to the ethical standards of the institutional review board and the 1964 Declaration of Helsinki and its later amendments. Written informed consent was obtained from each patient before radiotherapy. Patients who underwent SBRT between May 2022 and January 2023 for lung tumors 5 cm or smaller in size with respiratory motion greater than 10 mm and N0M0 disease were included; respiratory motion was measured using four-dimensional computed tomography (CT). Patients who had difficulty holding their breath for approximately 20 s and those with active interstitial pneumoniae were excluded.

### CT simulation

Prior to treatment planning, three to four FMs were implanted near the tumor using a bronchoscopy. The FMs were spherical Disposable Gold Marker (Olympus Medical Systems, Japan) with diameter of 1.5 mm. Reproducible BH measurements were conducted with the SDX system (DYN'R Medical Systems, France): this device monitors patient ventilation and displays it as a respiratory waveform in real time. Respiratory waveforms are fed back to the patient as visual data, and the patient voluntarily holds their breath on instruction from the medical staff. Patients were trained in the SDX system after receiving an overview of the device and method of holding their breath before the procedure was conducted. Treatment planning CT was performed 1 week after FM implantation using the SOMATOM Definition AS (Siemens Healthcare GmbH, Germany). The SDX system was used as recommended by the manufacturer, and patients were instructed to hold their breath at 85% of the maximum inspiratory volume. Actual inspiratory volumes ranged within a safety margin of approximately 100 mL. CT was performed three times using DIBH (IN-CT). To prepare for cases in which irradiation with DIBH could not be performed, we also performed CT with light expiration (EX-CT) so that, if necessary, we could quickly switch to RTRT, which is usually performed at our hospital. Treatment planning CT also confirmed that no implanted FMs had migrated.

### Treatment planning

The FM closest to the tumor was tracked during planning and treatment. The treatment planning system Eclipse (Varian Medical Systems, USA) was used for target delineation and dose calculations. Three IN-CT scans were fused: the scans were first registered using the bone structure and then registration was corrected using the FMs in the x-, y-, and z-axis directions. The gross tumor volume (GTV) was contoured for each dataset. A high correlation exists between the FM and tumor location during resting breathing^19,20^. Since this correlation may decrease with deep inspiration, a GTVsum was created by adding the GTVs for each IN-CT. The clinical target volume was defined as the GTVsum. The planning target volume (PTV) was determined by adding a 5-mm margin around the clinical target volume.

The treatment plan was created with six to seven ports using 6-MV flattening filter-free photons (DBRT-plan) and a dose rate of 1,400 MU/min. Eight patients had peripheral lesions and were prescribed 42 Gy in four fractions at the D95. The leaf margin was adjusted such that D2 was 125–130% of the prescribed dose. D95 and D2 were the doses administered to 95% and 2% of the PTV, respectively. The remaining patient had a central lesion and was prescribed 60 Gy in eight fractions to the isocenter of the PTV. The leaf margin was modified to cover the PTV with approximately 80% of the prescribed dose. The dose calculation algorithm used was Acuros External Beam (version 15.6.06). A similar treatment plan was created for EX-CT in cases for which DBRT could not be performed (conv-plan).

After treatment planning, the patient was placed on the treatment couch to ensure that the gantry did not touch the patient or SDX system. We confirmed that the FM could be tracked in all beam directions.

### Irradiation

CT was performed immediately before each irradiation session to confirm that no FM had migrated. Irradiation was performed using TrueBeam (Varian Medical Systems, USA). SyncTraX (Shimadzu Corporation, Japan) was used as the RTRT system: two sets of X-ray tubes are installed under the floor of the treatment room and image intensifiers are installed on the ceiling to provide real-time tracking of the FM near the tumor. The position of the FM is used as a surrogate for the position of the tumor. Irradiation is triggered when an FM enters a position within a 2-mm range in triaxial directions (a cube of 4 mm per side) of the position the FM held at the time of planning CT. Irradiation stops automatically when the FM is no longer in the gating box.

### Evaluation

Lung volumes were evaluated using IN-CT and EX-CT. Lungs were contoured automatically with Eclipse, and if the trachea and/or main bronchi were included, they were corrected manually. The left and right lungs were evaluated together. The ratio of lung volume on IN-CT to that on EX-CT was calculated. The mean lung dose, V20, V10, and V5 were calculated from dose-volume histograms: Vx was defined as the percentage of the lung volume that received x Gy. Comparisons between the lung doses in the two plans were made using the Wilcoxon signed-rank test. Correlations between lung volume and lung dose or pulmonary function test parameters were evaluated using the Spearman’s correlation coefficient.

**Figure 1:**
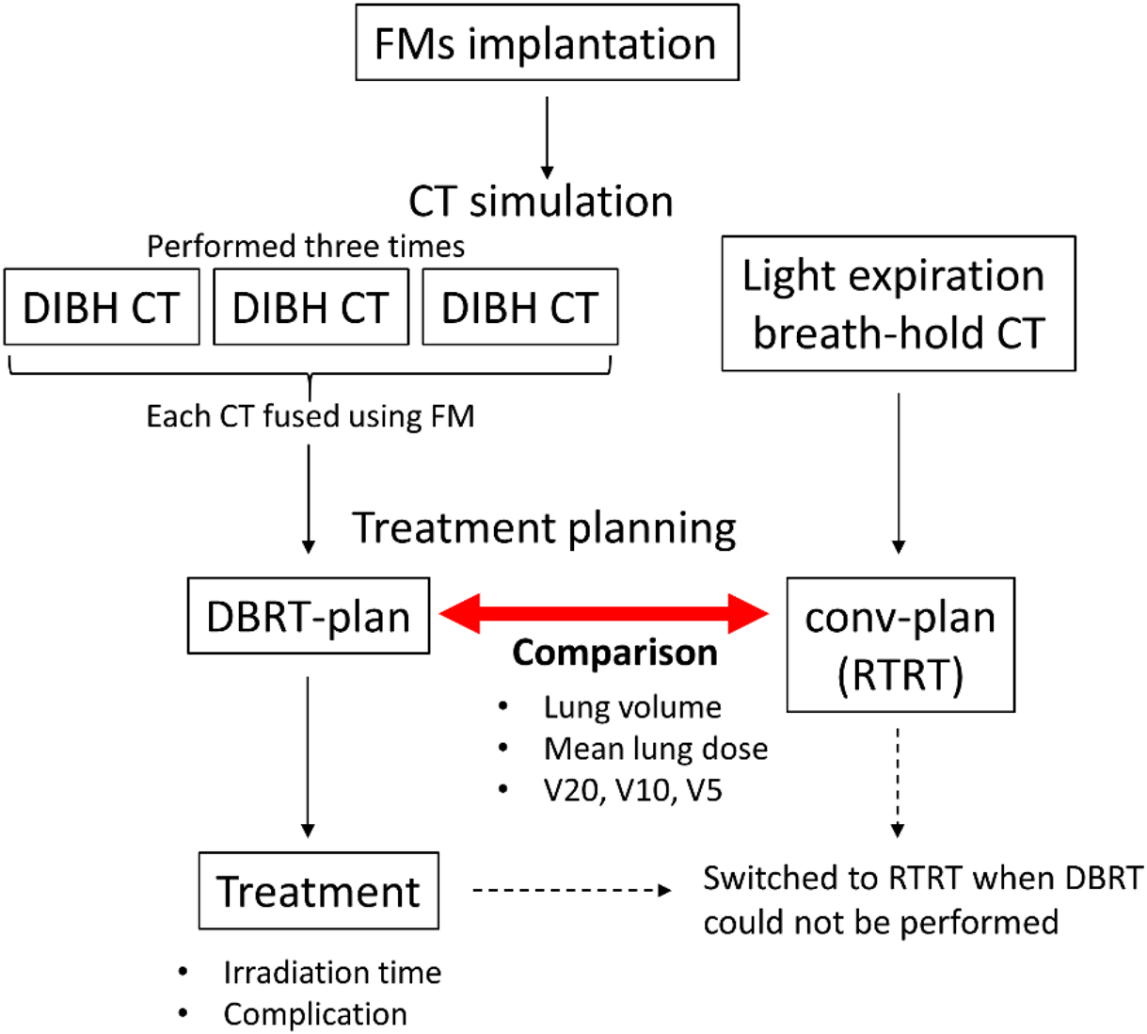
An overview of the method.

Irradiation time was defined as the time from the start of fluoroscopy for motion tracking after the patient was set up until the end of fluoroscopy. The ratio of irradiation time in the x-th session to that in the first session was calculated as the irradiation time ratio. An overview of the method is shown in Fig. 1.

A p value < 0.05 was considered to indicate a statistically significant difference.

### Ethical approval and consent to participate

All procedures performed in studies involving human participants were in accordance with the ethical standards of the institutional and/or national research committee and with the 1964 Declaration of Helsinki and its later amendments or comparable ethical standards. The study design was approved by the ethics committee of Yamaguchi University Hospital (approval number 2022-077). All patients provided written informed consent for the use of their data in clinical research before the administration of radiotherapy and had the opportunity to opt out of the study.

## Results

Nine patients were enrolled in this study. Patient characteristics are shown in Table 1 and supplementary Table 1. The median age of the patients was 75 (range 70–82) years. Six (66.7%) patients were male and three (33.3%) were female. Eight (88.9%) patients had lesions in the lower lobe, and the remaining patient had a lingual segment. Four (44.4%) patients had a history of lung surgery: patient 1 had undergone two surgeries for lung tumors, both on the contralateral side; patient 5 had a history of ipsilateral surgery in the upper lobe; patients 7 and 8 had a history of contralateral lung tumor surgery; and patient 7 had a history of ipsilateral SBRT to another lobe. None of the other patients had a history of radiation therapy to the chest. Eight (88.9%) patients were smokers. All patients underwent pulmonary function tests before undergoing CT simulation. On an average, 28 (range 10–203) days lapsed between the pulmonary function test and the CT simulation.

**Table 1:** Patient characteristics.

Patient number	Sex	Age, years	Location	PS	History of surgery	History of RT	VC, L	IC, L	IRV, L	%FEV_1_	PTV, mL	LV EX, L	LV IN, L	Ratio of LV
1	Female	75	RLL	0	Yes	No	2.60	1.69	1.17	97.7	12.4	1984.9	3814.3	1.92
2	Male	75	LLL	1	No	No	2.96	2.32	1.65	50.8	26.2	3404.0	4758.4	1.40
3	Male	73	LLL	0	No	No	3.77	2.18	1.67	46.6	21.2	5318.8	6647.5	1.25
4	Male	70	LLL	0	No	No	2.80	1.89	1.35	88.6	5.2	2369.3	3820.0	1.61
5	Male	81	LLL	0	Yes	No	4.17	2.87	1.85	110.2	10.7	2872.2	5241.8	1.83
6	Male	74	LLL	1	No	No	3.08	2.50	1.94	87.5	120.6	2605.7	4483.0	1.72
7	Female	82	LUL (lingular)	0	Yes	Yes	2.07	1.65	1.10	112.8	10.7	1644.9	2833.5	1.72
8	Male	75	LLL	0	Yes	No	3.84	1.38	0.69	82.7	11.0	3645.5	5189.5	1.42
9	Female	73	RLL	0	No	No	2.25	1.18	0.66	69.3	9.0	3184.8	4514.3	1.42

RLL, right lower lobe; LLL, left lower lobe; LUL, left upper lobe; PS, performance status; RT, radiotherapy; VC, vital capacity; IC, Inspiratory capacity; IRV, inspiratory reserve volume; %FEV_1_, forced expiratory volume in 1 s; PTV, planning target volume; LV, lung volume; EX, expiration; IN, inspiration.

DBRT was completed for all patients. The median distance from the tumor center to the marker center was 1.46 (range 0.44–2.66) cm, and the median PTV was 11.0 (range 5.2–120.6) mL. The lung volumes and doses on EX-CT and IN-CT are shown in Table 2. The mean dose, V20, and V10 were significantly lower in the DBRT-plan than in the conv-plan (p = 0.003, 0.003, and 0.003, respectively). No significant differences were observed in V5 (p = 0.074).

**Table 2:** Median (range) lung volumes and doses on expiration and inspiration computed tomography.

	Expiration (range)	Inspiration (range)	Mean rate of change, % (± SD)	p value
Volume, mL	2872.2 (1644.9–5318.8)	4514.3 (2833.5–6647.5)	58.8 (± 22.6)	0.003
Mean dose, Gy	1.44 (1.25–5.62)	1.41 (1.09–4.63)	− 14.0 (± 12.0)	0.003
V20, %	1.49 (0.74–6.29)	1.01 (0.62–4.12)	− 27.6 (± 13.9)	0.003
V10, %	3.92 (2.31–17.51)	3.23 (2.05–13.25)	− 19.1 (± 12.2)	0.003
V5, %	7.94 (6.17–26.64)	8.10 (6.50–24.86)	− 9.5 (± 14.7)	0.074

Vx, the volumes of the lung that received x Gy. SD, standard deviation.

The ratio of lung volume was significantly correlated with the percentage of forced expiratory volume in 1 s (%FEV_1_) (ρ = 0.917, p = 0.001; supplementary Fig. 1). A strong negative correlation was observed between the ratio of lung volume and lung volume on EX-CT (ρ = − 0.800, p = 0.013; supplementary Fig. 2).

Lung volume on EX-CT and the decrease in the mean lung dose showed a strong negative correlation (ρ = − 0.850, p = 0.006; supplementary Fig. 3). Similarly, lung volume on EX-CT had a strong negative correlation with the decrease in both V20 and V10 (ρ = − 0.800, p = 0.013 and ρ = − 0.833, p = 0.008, respectively; supplementary Figs. 4 and 5). No significant correlation was observed between lung volume on EX-CT and the decrease in V5 (ρ = − 0.617, p = 0.086; supplementary Fig. 6).

A positive correlation was observed between the ratio of lung volume and the decrease in mean lung dose or V20 (ρ = 0.648, p = 0.049 and ρ = 0.879, p = 0.019, respectively; supplementary Figs. 7 and 8). No significant correlation was found between the ratio of lung volume and the decrease in V10 or V5 (ρ = 0.624, p = 0.060 and ρ = 0.297, p = 0.407, respectively; supplementary Figs. 9 and 10).

**Figure 2:**
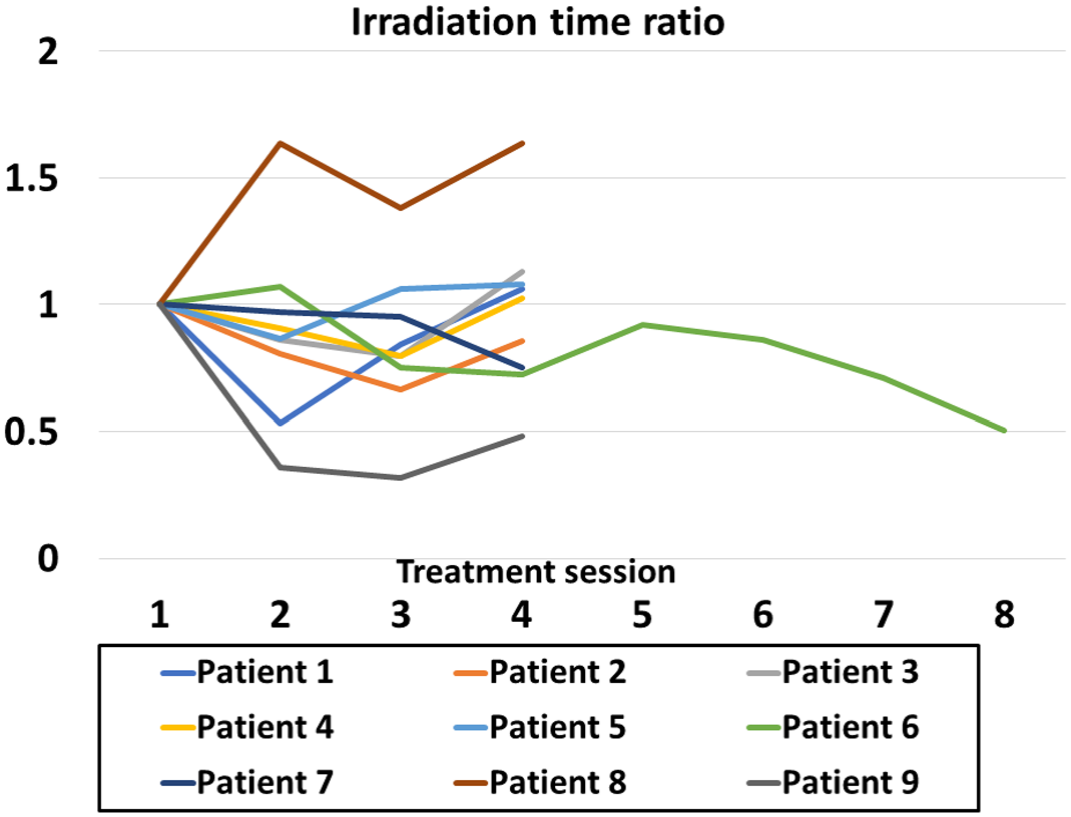
For each patient, the ratio of the second and subsequent irradiation times to the first irradiation time generally remained unchanged.

The average irradiation time for one session was 31 (range 25–49) min. Figure 2 shows the irradiation time ratio for each patient.

## Discussion

**Figure 3:**
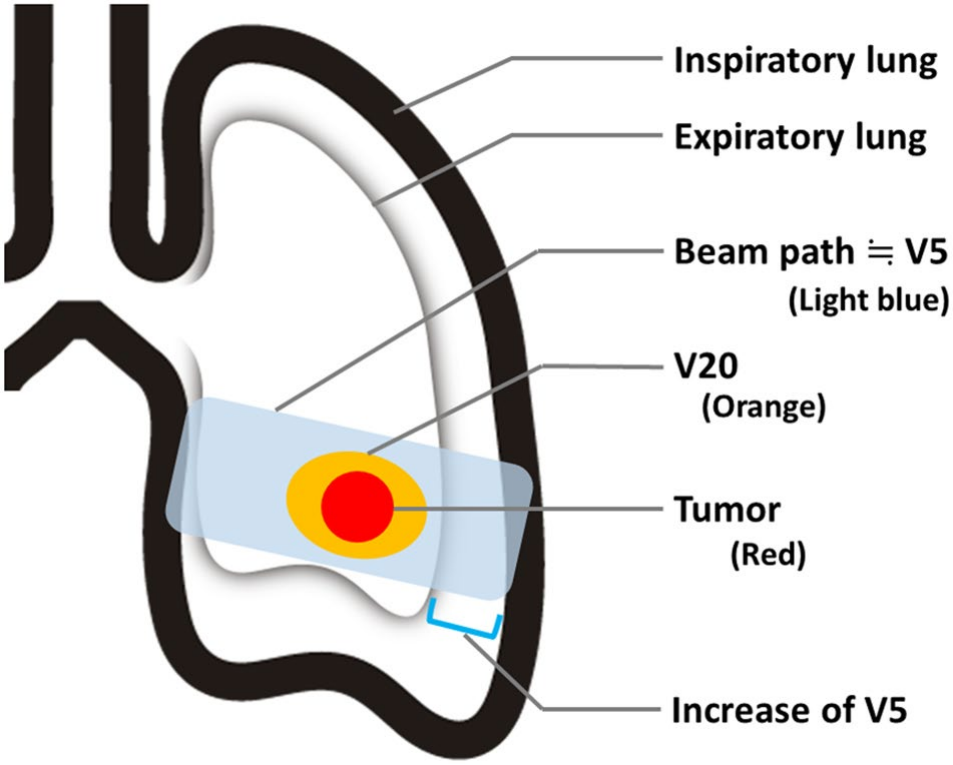
With deep inspiration breath hold, as the lungs expand, the distance and volume that X-rays pass through in the lungs also increase, making it difficult to reduce the low-dose area.

In this study, the increased lung volume, made possible with DBRT, enabled a significant decrease in the mean lung dose, V20, and V10. This is the greatest advantage of irradiation using DIBH. However, there was no significant difference in V5 between the DBRT-plan and conv-plan. The area irradiated with more than 20 or 10 Gy was relatively limited around the PTV, but the area irradiated with more than 5 Gy included most of the area through which the beam passed. Therefore, when DIBH is used, the distance and volume that X-rays pass through also increase, making it difficult to achieve a reduction in the low-dose area (Fig. 3).

The ratio of lung volume significantly correlated with %FEV_1_. %FEV1 is a value used for staging chronic obstructive pulmonary disease (COPD), and low %FEV1 indicates advanced disease^21^; that is patients with severe COPD tend to have a low ratio of lung volume. A strong negative correlation was also observed between the ratio of lung volume and lung volume on EX-CT. Patients with a large lung volume on EX-CT are less likely to have further infiltration. As COPD progresses, adequate expiration becomes difficult and expiratory lung volume increases^22^. Thus, patients with COPD are unlikely to achieve further volume expansion.

A negative correlation was found between the lung volume on EX-CT and the decrease in mean lung dose, V20, or V10, with a positive correlation observed between the ratio of lung volume and the decrease in mean lung dose or V20. An increase in lung volume because of deep inspiration produces a relative decrease in the area exposed to moderate or high doses. As data accumulation and analysis progress, clinicians may be able to use lung volume to determine whether irradiation with DIBH is beneficial for patients before treatment planning.

**Figure 4:**
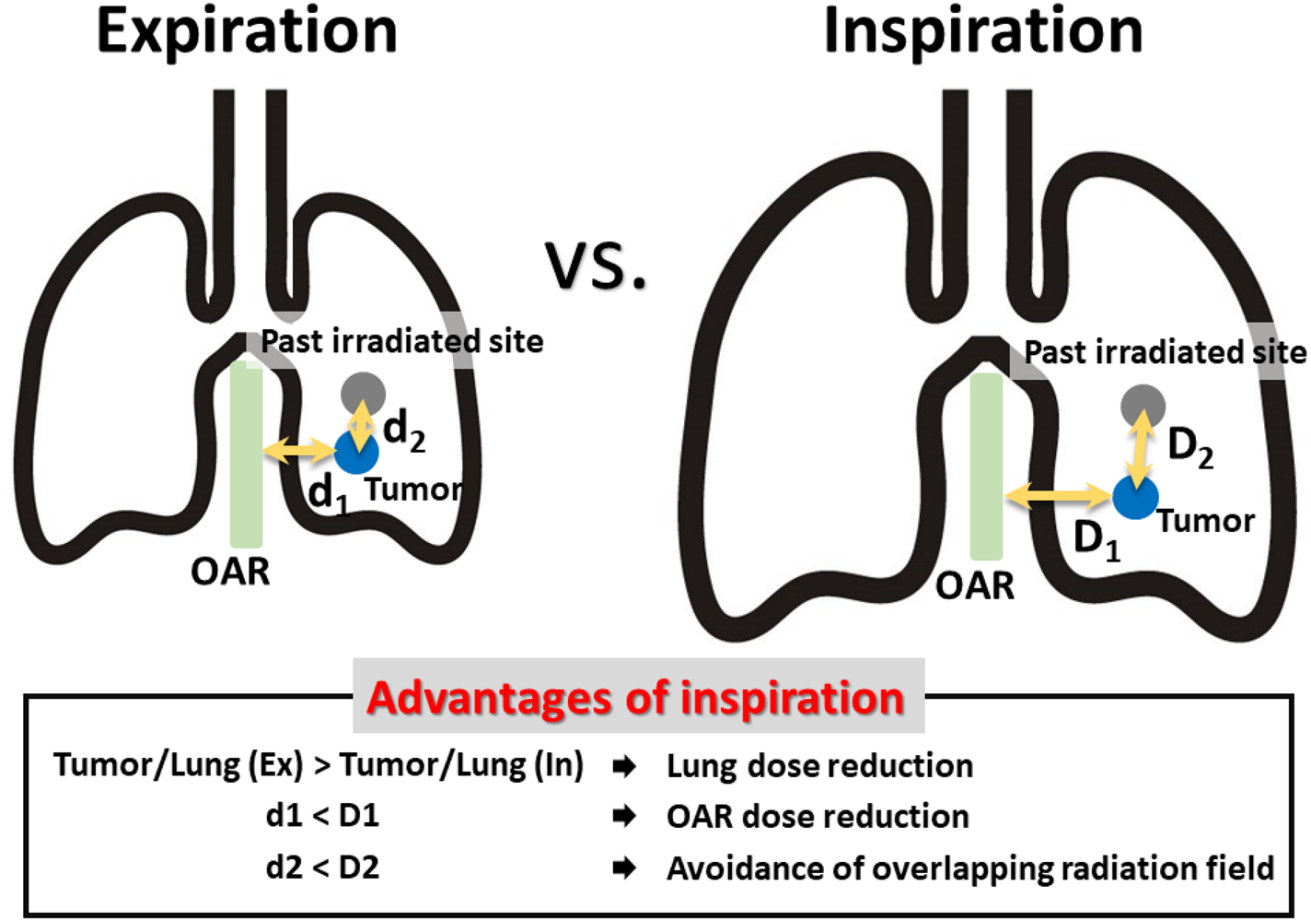
Radiotherapy with deep inspiration breath hold reduces the dose the lung and organs at risk are irradiated with. This technique allows for the distance between irradiated sites to be increased in patients with a history of irradiation. D1, d1: distance between tumor and organ at risk. D2, d2: distance between tumor and past irradiated site.

The volume of the lungs changes considerably respiration. As lung volume increases with inspiration, the relative proportion of tumors in the lung decreases, and as a result, radiotherapy with DIBH can use a lower dose than radiotherapy with expiration (Fig. 4).

In addition, the expansion of the lungs increases the distance between the tumor and the organs at risk (OARs) such as the spinal cord, esophagus, bronchi, and heart; thus, the distance is increased between the OAR and the irradiation field, thereby reducing the doses to these organs. For patients with a history of radiotherapy to the lung, the distance from the previously irradiated site can also be maintained, thereby reducing the possibility of irradiation fields and areas overlapping and increasing the safety of the procedure. Despite the many advantages of performing SBRT for lungs with DIBH, it is often performed during expiration^13^. Radiotherapy is not performed with DIBH because of the type of RMM it is. With the BH method, inhalation is less stable than exhalation and reproducibility of tumor location is poorer during inspiration than during expiration^11,12^. Nearly half of the cases reported in the literature are ineligible for irradiation with DIBH owing to reproducibility issues^7,13^. If the variation in the tumor location for each BH is large, the range of variation is included in the target, which leads to an expansion of the irradiation field. In SBRT, a large dose is administered in one session, making expansion of the irradiation field undesirable. In addition, DIBH is an unfavorable RMM technique in RTRT because tumors move faster during inspiration than during expiration^17^. In RTRT, the beam is turned on when the marker enters the gating box, but a slight time lag occurs between when the beam-on signal is issued and when the beam is actually turned^18^. The faster the tumor moves, the larger the distance it moves during this time lag. Similarly, there is a slight time lag when the beam, triggered by the marker leaving the gating box, is turned off. A margin for this beam-on and beam-off delay, is provided, but the margin inevitably becomes large for inspiration in cases with a high tumor velocity. Moreover, with free breathing, the reproducibility of the intrafractional positioning is higher at end of expiration than at end of inspiration^23^. Therefore, irradiation is aimed to occur at the end of expiration when movement is the slowest and reproducibility is the highest in the RTRT^24^. We developed and successfully performed DBRT, in which RTRT is performed during DIBH. DBRT addresses all these problems. The high reproducibility of the tumor location during DIBH is guaranteed by tracking the FM, which is an internal respiration signal in real-time, and using it as a beam-on trigger. The beam is not turned on if the location has not been reproduced. Although previous attempts have been made to perform SBRT during DIBH using an external respiration signal^25–28^, few reports exist on the use of SBRT for lung tumors that utilize DIBH with real-time tracking of internal respiration signals^29^. Concerns about errors in positional correlation between the FM and tumor are addressed by summing the GTV in three IN-CTs with marker-based registration. Extremely poor reproducibility of the tumor location during DIBH raises the concern that the beam will not turn on and irradiation will not end. We used an SDX system to improve the reproducibility of the tumor location during DIBH and to increase the frequency of beam-on. All patients in this study could complete all treatment sessions with DBRT. Theoretically, the spirometer-based BH method, as well as other devices that support BH, can be used.

DBRT can resolve two additional problems. First, during irradiation with BH, “residual motion,” in which the tumor moves slowly despite BH, is problematic^30^. Even if the tumor is within the irradiation field at the start of irradiation, a risk exists that residual motion will cause the tumor to move out of the field during irradiation. Even if residual motion occurs during DBRT, irradiation stops when the FM moves out of the gating box. Thus, missed hits arising from residual motion are avoided. Second, sudden tumor motion may be problematic, for example, when a patient cannot maintain BH and exhales or when there is body movement for some reason. If the beam is manually turned on and off without gating with the respiratory waveform, detecting these abnormalities and turning the beam off will take time. This may impact tumor control because a missed hit to the tumor will occur. With DBRT, the beam is turned off as soon as the FM leaves the gating box. In summary, the advantages of DBRT include: reduction in lung dose due to larger lung volume, higher accuracy of the RTRT system with a tracking error of < 0.4 mm^16^, reduction on OAR dose due to a greater distance to this OAR, and detection ability of residual motion (Table 3).

**Table 3:** Comparison between the DBRT and existing SBRT methods.

	SBRT during expiration breath hold	SBRT during DIBH (without FMs)	RTRT	DBRT
FM implantation			Need	Need
Reproducibility of tumor position	Fair		Good	Good
Reduction in lung dose		Fair		Good
Distance from OAR		Good		Good
Residual motion	Not monitored	Not monitored	Recognizable	Recognizable

DBRT, deep inspiration breath hold real-time tumor-tracking radiation therapy; SBRT, stereotactic body radiation therapy; DIBH; deep inspiration breath hold; FM, fiducial marker; RTRT, real-time tumor-tracking; OAR; organs at risk.

A disadvantage of DBRT is that it requires a long irradiation time: on average, 31 min for each session. Patients with the longest irradiation times required an average of 49 min, with some sessions lasting up to 57 min. The average irradiation time for conventional RTRT at our hospital, which irradiates the terminal expiration, is approximately 19 min. Checking for FM migration, setting up the patient, and moving the gantry and couch add to the time spent for a DBRT session. The ratio of the second and subsequent irradiation times to the first irradiation time for each patient was expected to gradually shrink, but in practice it generally remained unchanged. Regarding the conventional RTRT, fluoroscopy is always on due to performing treatment under free breath. Using the DBRT, fluoroscopy is turned on only during breath hold by patients as instructed by the medical staff. The majority of irradiation time is spent regulating patient's breathing after breath hold, whereas only a small amount of the time is actually spent for breath hold. Therefore, the time when fluoroscopy is turned on is almost as the same as that using the conventional method.

This study was limited by its retrospective nature, small sample size, and lack of clinical assessment of adverse events and treatment outcomes. The appropriate sample size was estimated to be 37 cases with an alpha error of 0.05 and power of 0.80. Future research that uses a larger sample size and includes an analysis of follow-up data to evaluate the clinical assessment is needed.

## Conclusions

We developed DBRT, a SBRT that is administered using the DIBH technique. It combines a spirometer-based BH support system with an RTRT system. All patients who underwent DBRT completed the procedure without any complications. DBRT is a promising method of irradiation that can significantly reduce lung doses. The long irradiation time that this method uses is an issue that requires future research.

### Supplementary Information

Supplementary Information.

## Data Availability

The datasets used and/or analysed during the current study are available from the corresponding author on reasonable request.

## Abbreviations

RMM: Respiratory motion management
SBRT: Stereotactic body radiation therapy
BH: Breath hold
RTRT: Real-time tumor-tracking radiation therapy
FM: Fiducial marker
DIBH: Deep inspiration breath hold
DBRT: Deep inspiration breath hold real-time tumor-tracking radiotherapy
CT: Computed tomography
GTV: Gross tumor volume
PTV: Planning target volume
COPD: Chronic obstructive pulmonary disease
