# Supplementary material for: Deep inspiration breath hold real-time tumor-tracking radiation therapy (DBRT) as a novel stereotactic body radiation therapy approach for lung tumors
Source: Sci Rep. 2024 Jan 29;14:2400. doi: 10.1038/s41598-024-53020-4 (PMC10825222; doi:10.1038/s41598-024-53020-4)
Supplement: Supplementary file 1 — Supplementary Information. [file 41598_2024_53020_MOESM1_ESM.docx]

| **Supplementary table 1.** Patient characteristics (N = 9) | | |  |
| --- | --- | --- | --- |
|  |  | N (%) |  |
| Age | Median (range) (years) | 75 (70–82) |  |
| Sex | Male / female | 6 (66.7) / 3 (33.3) |  |
| Performance status | 0 / 1 | 7 (77.8) / 2 (22.2) |  |
| Location | Left lower lobe | 6 (66.7) |  |
|  | Right lower lobe | 2 (22.2) |  |
|  | Left upper lobe (lingular) | 1 (11.1) |  |
| Primary / metastasis |  | 8 (88.8) / 1 (11.1) |  |
| History of surgery | Yes / no | 4 (44.4) / 5 (55.6) |  |
| History of chest RT | Yse / no | 1 (11.1) / 8 (88.9) |  |
| Smoking | Current / past / never | 0 (0) / 8 (88.9) / 1 (11.1) |  |
| VC | Median (range) (L) | 2.96 (2.07–4.17) |  |
| IC | Median (range) (L) | 1.89 (1.18–2.87) |  |
| IRV | Median (range) (L) | 1.35 (0.66–1.94) |  |
| %FEV_1_ | Median (range) (%) | 87.5 (46.6–112.8) |  |
| VC, vital capacity; IC, inspiratory capacity; IRV, inspiratory reserve volume; %FEV_1_, the percentage of forced expiratory volume in one second | | |  |
|  |  |  |  |


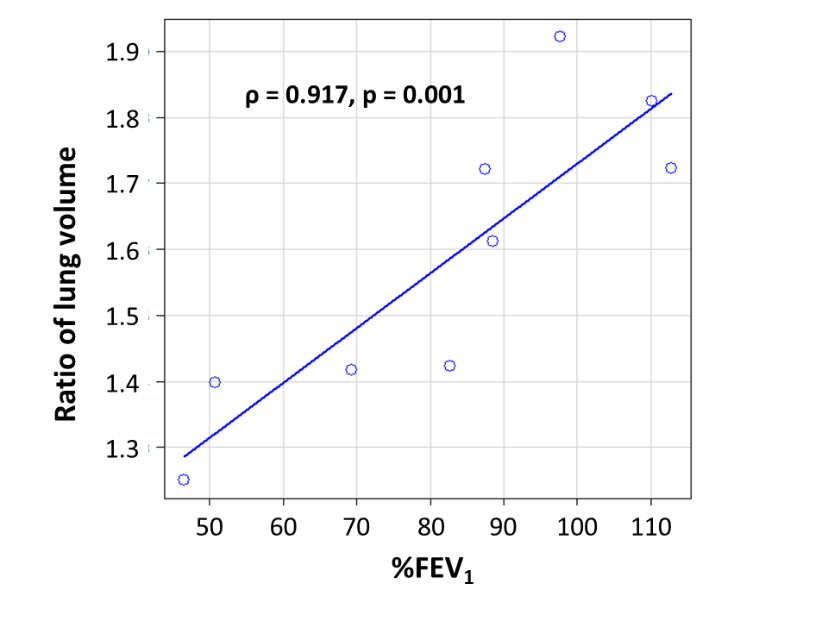
**Supplementary figure 1.** The ratio of lung volume was significantly correlated with the %FEV_1_. %FEV_1_, percentage of forced expiratory volume in 1 second.


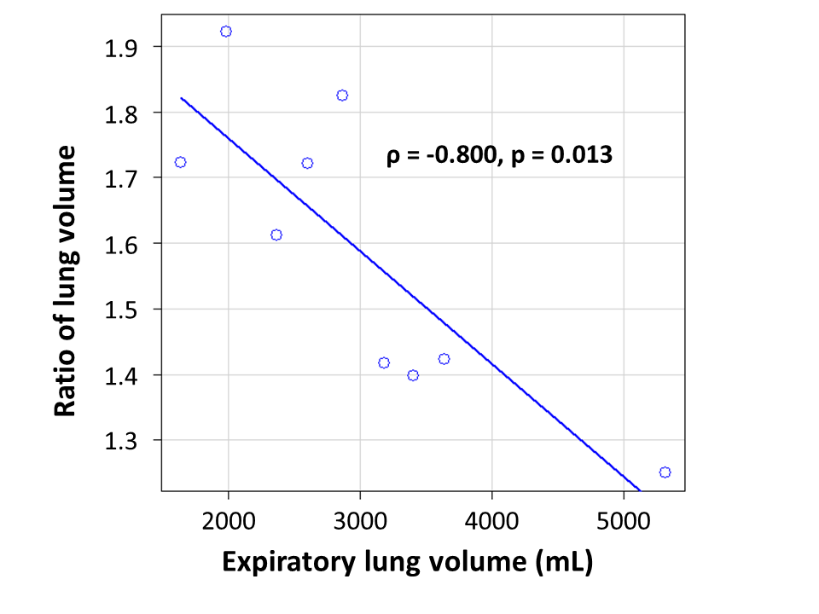
**Supplementary figure 2.** A strong negative correlation was observed between the ratio of lung volume and lung volume on expiratory CT.


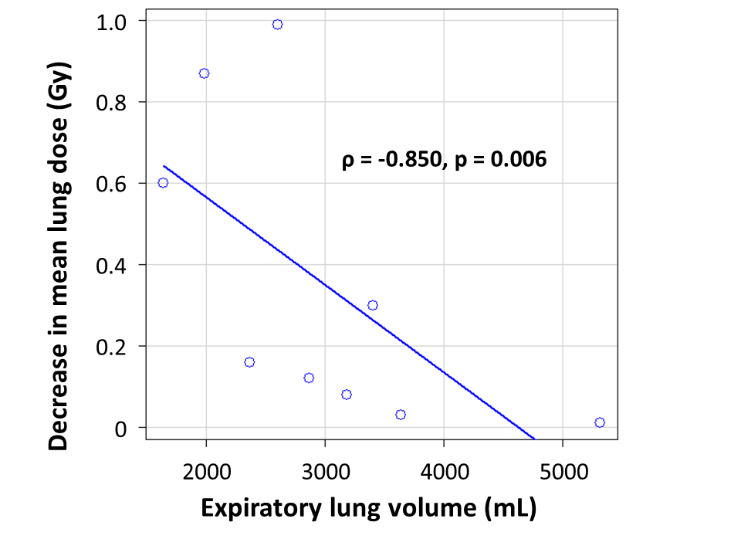
**Supplementary figure 3**. Lung volume on expiratory CT and the decrease in the mean lung dose showed a strong negative correlation.


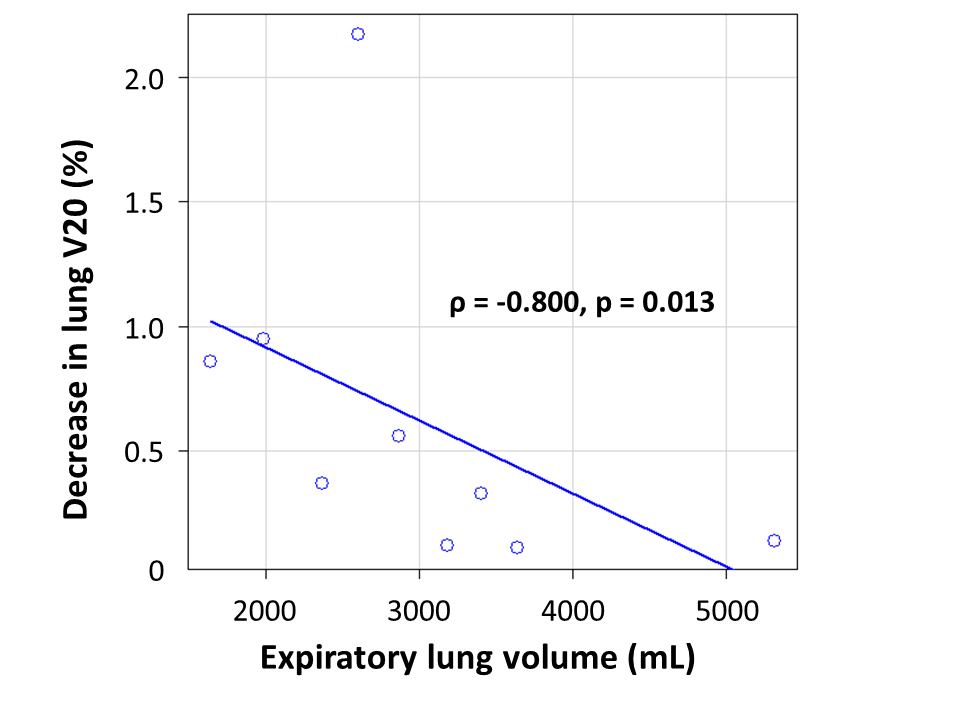
**Supplementary figure 4.** Lung volume on expiratory CT had a strong negative correlation with the decrease in V20.


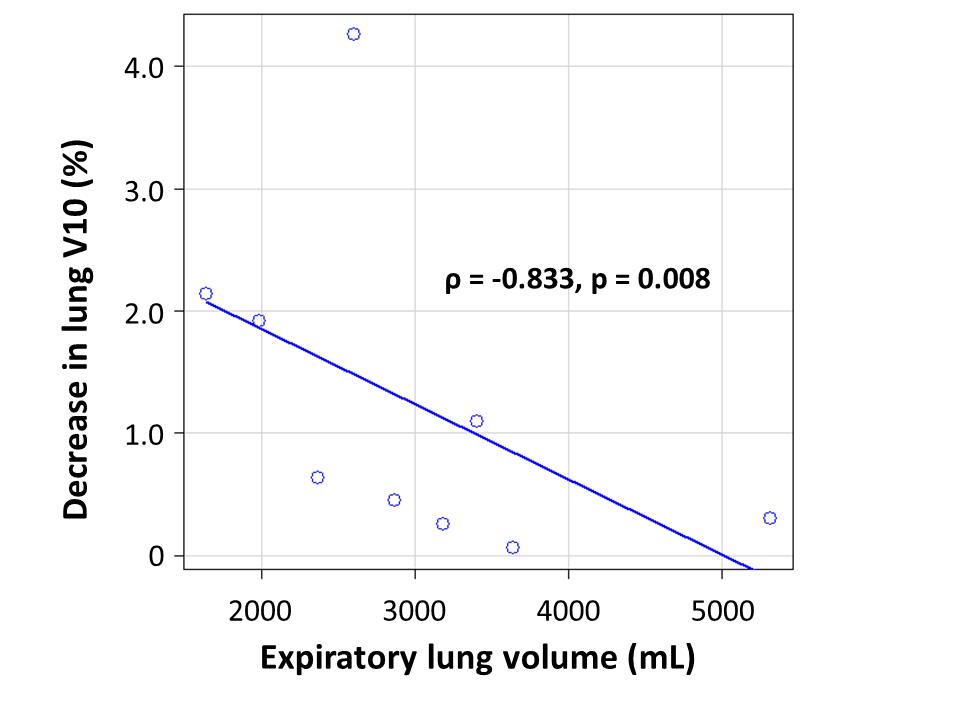
**Supplementary figure 5.** Lung volume on expiratory CT had a strong negative correlation with the decrease in V10.


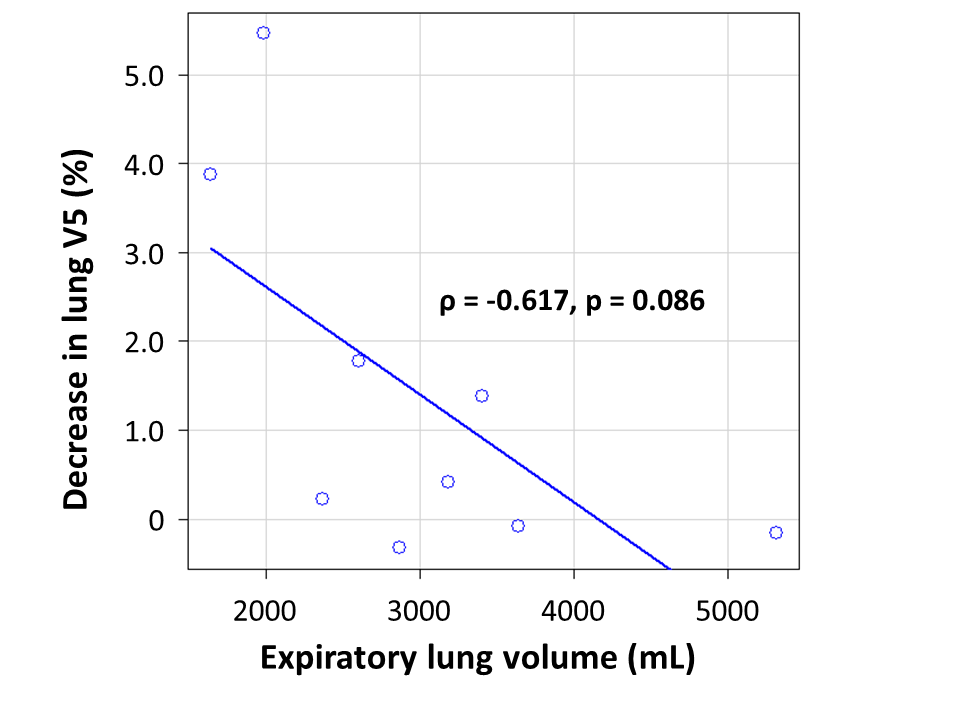
**Supplementary figure 6.** No significant correlation was observed between lung volume on expiratory CT and the decrease V5.


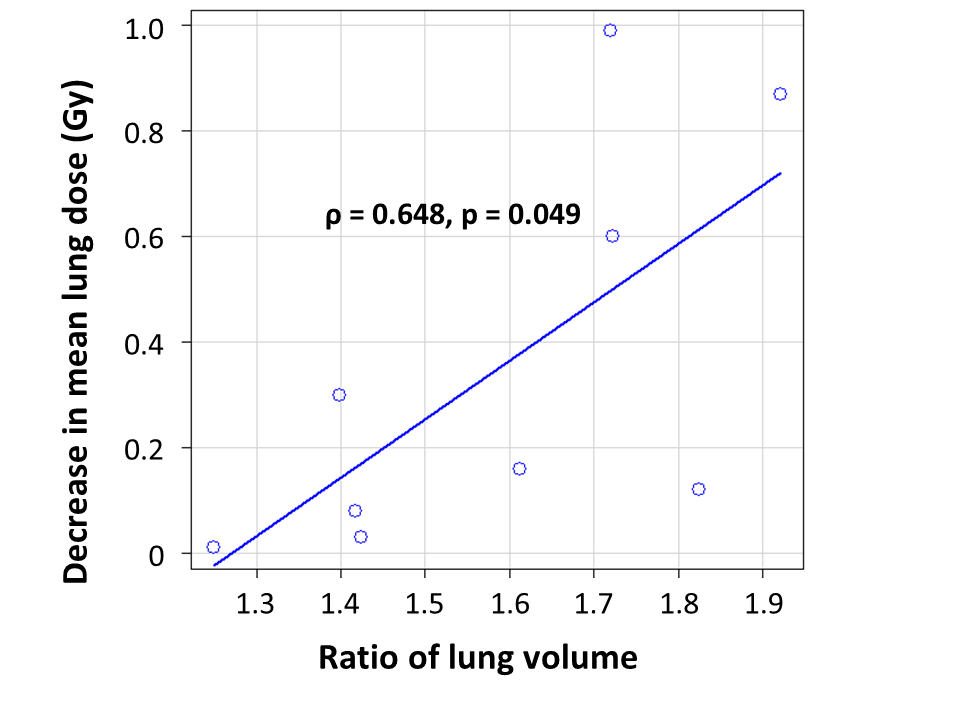
**Supplementary figure 7.** A positive correlation was observed between the ratio of lung volume and the decreased in mean lung dose.


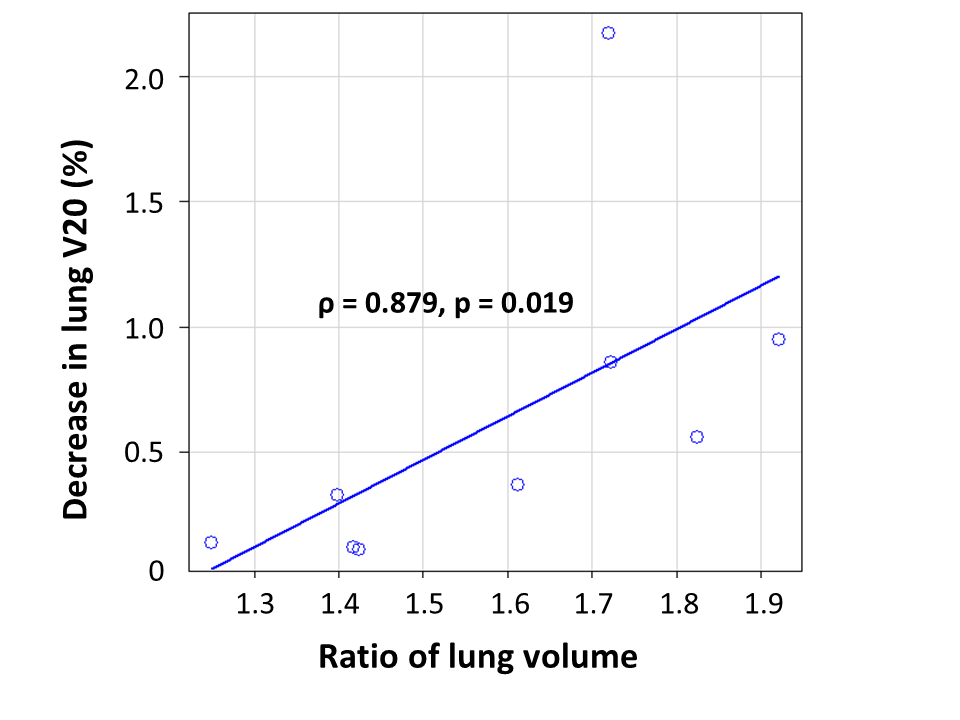
**Supplementary figure 8.** A positive correlation was observed between the ratio of lung volume and the decreased in V20.


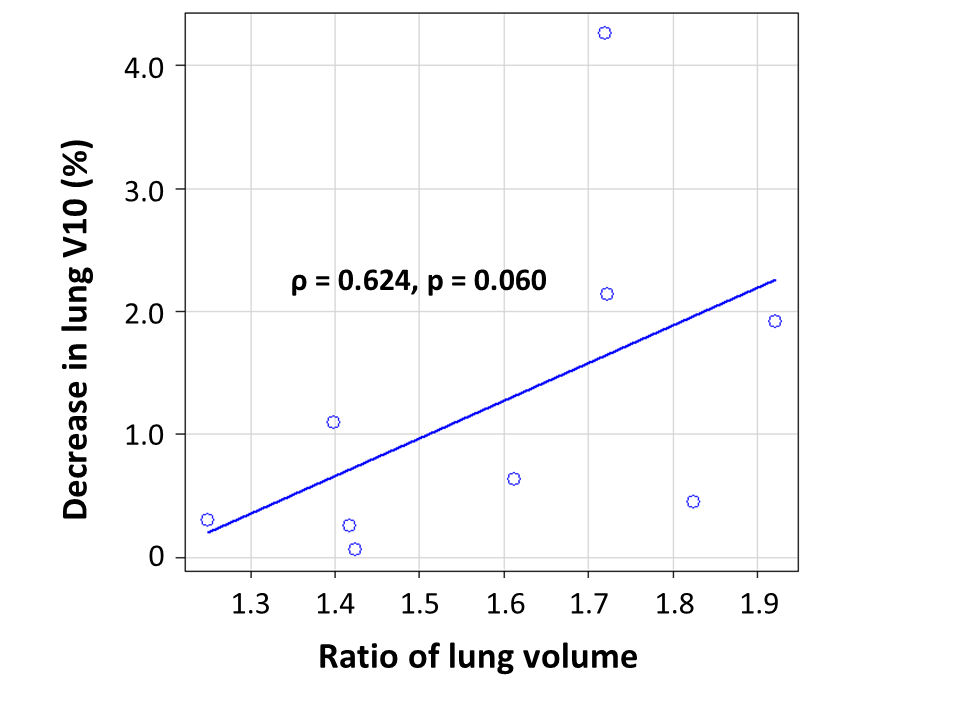
**Supplementary figure 9.** No significant correlation was found between the ratio of lung volume and the decrease in V10.


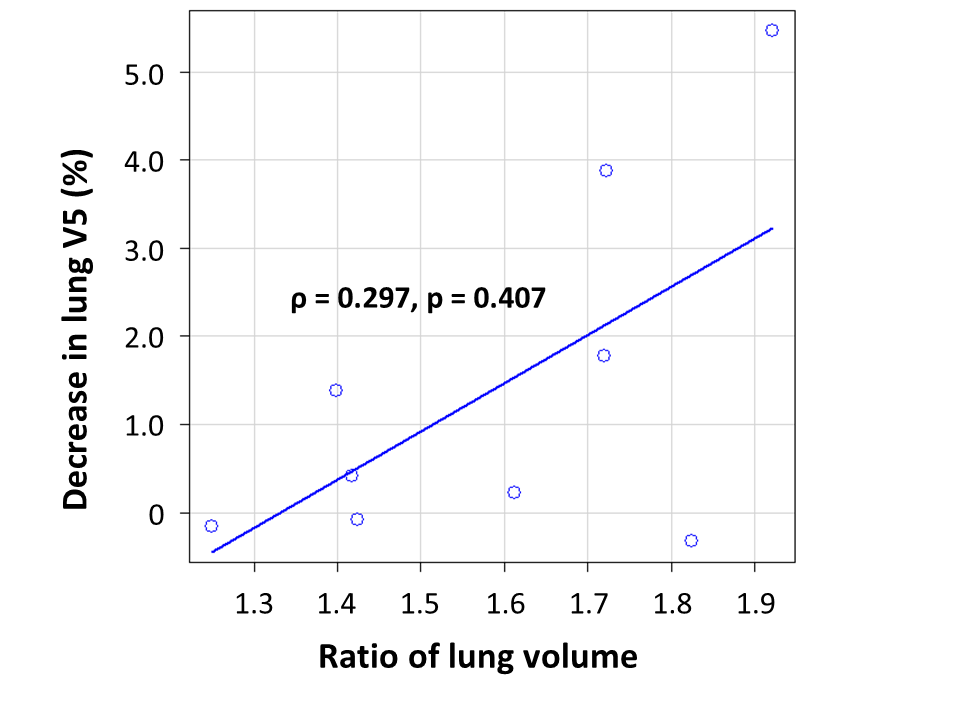
**Supplementary figure 10.** No significant correlation was found between the ratio of lung volume and the decrease in V5.
